# Model-informed COVID-19 exit strategy with projections of SARS-CoV-2 infections generated by variants in the Republic of Korea

**DOI:** 10.1186/s12889-022-14576-w

**Published:** 2022-11-17

**Authors:** Sung-mok Jung, Kyungmin Huh, Munkhzul Radnaabaatar, Jaehun Jung

**Affiliations:** 1 Kyoto University School of Public Health, Yoshidakonoe cho, Sakyo ku, Kyoto city, 6068501 Japan; 2 Graduate School of Medicine, Hokkaido University, Kita 15 Jo Nishi 7 Chome, Kita-ku, Sapporo-shi, Hokkaido 060-8638 Japan; 3 Division of Infectious Diseases, Department of Medicine, Samsung Medical Center, Sungkyunkwan University School of Medicine, Seoul, 06351 South Korea; 4 Artificial Intelligence and Big-Data Convergence Center, Gil Medical Center, Gachon University College of Medicine, Incheon, 21565 South Korea; 5 Department of Preventive Medicine, Gachon University College of Medicine, Incheon, 21565 South Korea

**Keywords:** COVID-19, Exit strategy, Projections, Mathematical modelling, The Republic of Korea

## Abstract

**Background:**

With the prompt administration of coronavirus disease 2019 (COVID-19) vaccines, highly vaccinated countries have begun to lift their stringent control measures. However, considering the spread of highly transmissible new variants, resuming socio-economic activities may lead to the resurgence of incidence, particularly in nations with a low proportion of individuals who have natural immunity. Here, we aimed to quantitatively assess an optimal COVID-19 exit strategy in the Republic of Korea, where only a small number of cumulative incidences have been recorded as of September 2021, comparing epidemiological outcomes via scenario analysis.

**Methods:**

A discrete-time deterministic compartmental model structured by age group was used, accounting for the variant-specific transmission dynamics and the currently planned nationwide vaccination. All parameters were calibrated using comprehensive empirical data obtained from the Korea Disease Control and Prevention Agency.

**Results:**

Our projection suggests that tapering the level of social distancing countermeasures to the minimum level from November 2021 can efficiently suppress a resurgence of incidence given the currently planned nationwide vaccine roll-out. In addition, considering the spread of the Delta variant, our model suggested that gradual easing of countermeasures for more than 4 months can efficiently withstand the prevalence of severe COVID-19 cases until the end of 2022.

**Conclusions:**

Our model-based projections provide evidence-based guidance for an exit strategy that allows society to resume normal life while sustaining the suppression of the COVID-19 epidemic in countries where the spread of COVID-19 has been well controlled.

**Supplementary Information:**

The online version contains supplementary material available at 10.1186/s12889-022-14576-w.

## Background

The coronavirus disease 2019 (COVID-19) pandemic has challenged the resilience of healthcare systems around the world, resulting in enormous casualties and economic burdens [1]. In response, a variety of non-pharmaceutical interventions (NPIs), which are seen as important tools in tackling the growing COVID-19 epidemic, have been implemented in many of the heavily affected areas [2]. Despite the low number of reported COVID-19 cases and deaths in the Republic of Korea (hereafter “Korea”), the widespread of severe acute respiratory syndrome coronavirus 2 (SARS-CoV-2) has also had a significant impact on the country. Korea is one of the few countries in the world to have successfully controlled the SARS-CoV-2 transmissions by implementing the “3 T strategy” (i.e., the response strategy based on tracing, testing, and treating), along with a social distancing scheme without a lockdown and mask-wearing [3]. Such broad restrictions on people’s lives have enabled keeping a low prevalence of COVID-19 in Korea, however, its socio-economic impacts have not been negligible. For instance, all students of elementary, middle, and high school were subjected to online classes, and restaurants and bars were asked to curtail their operational hours following the level of social distancing countermeasures (Fig. S1 and Table S1; see Additional file) [4]. Thus, as the proportion of individuals with a second dose of vaccine had reached 70% of the total population [5], the national government announced an exit strategy with a gradual return to normal life beginning in November 2021 to minimize the socio-economic burdens of the disease [6].

However, given the rapid spread of highly transmissible new SARS-CoV-2 variants (i.e., the Alpha and Delta variants), resuming socio-economic activities without proper preparation, even after nationwide vaccination, may lead to a significant resurgence of hospitalizations and deaths in Korea. Moreover, the need for a tailored exit strategy can be more highlighted in countries with a low cumulative COVID-19 incidence like Korea. In this respect, a mathematical modelling approach can play a critical role in identifying an optimal exit strategy allowing us to taper the impact of COVID-19 [7]. In addition, while uncertainties in the empirical data (e.g., under-ascertainment) make long-term forecasting of the COVID-19 epidemic challenging [8], Korea was able to collect an enormous amount of precise empirical data through active contact tracing and testing. Thus, with the comprehensive epidemiological data of Korea, along with detailed information on the proportion by variant and administered COVID-19 vaccines [9–11], mathematical modelling can provide substantial insights into the transmission dynamics of new SARS-CoV-2 variants and the impacts of prospective exit strategies on it. Previous studies employed the mathematical modelling approach to explore the future SARS-CoV-2 waves in Korea following behavior changes, however, such studies were unable to adequately account for the potential effects of highly transmissible new variants [12, 13]. Here, we aim to characterize an optimal COVID-19 exit strategy in Korea, using a mathematical modelling framework integrating the transmission dynamics of SARS-CoV-2 by variant and the currently planned nationwide vaccination.

## Methods

### Empirical dataset in Korea

#### Epidemiological data

**Fig. 1:**
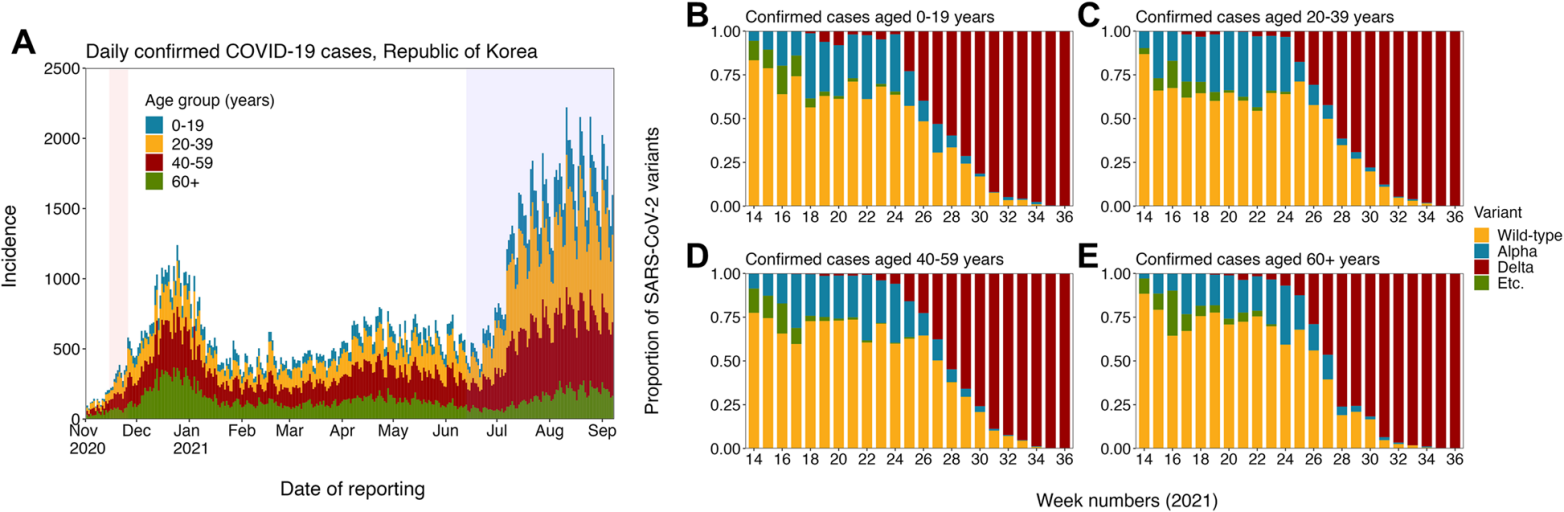
Epidemic curve of COVID-19 and the proportion of SARS-CoV-2 variants among tested samples in the Republic of Korea. **A** Epidemic curve of age-stratified confirmed COVID-19 cases by date of reporting (1 November 2020–8 September 2021) and **B-E** the proportion of SARS-CoV-2 variants among tested samples by age group (week 14–36 in 2021). Both data were retrieved from non-publicly available reports from the Korea Disease Control and Prevention Agency, and all SARS-CoV-2 variants were categorized into four types: Wild-type, Alpha variant, Delta variant, and the other variants (Etc.)

Two types of epidemiological data were used in our study: “incidence data” and “variant data.” The incidence data includes information on confirmed COVID-19 cases reported from 20 January 2020 to 8 September 2021 (Fig. 1A), while the variant data comprises the weekly incidence by variant among tested samples during the corresponding period (Fig. 1B–E). Of the 265,423 cases in the incidence data, 42,727 (16%) were tested (the variant data) and categorized into four types of variant: wild-type variant (i.e., the dominant circulating SARS-CoV-2 variant in Korea), Alpha variant, Delta variant, and the other variants. Both datasets were retrieved from non-publicly available reports provided by the Korea Disease Control and Prevention Agency (KDCA), and all confirmed cases were aggregated into the following four age groups: individuals aged 0–19 years, 20–39 years, 40–59 years, and 60 years and older.

#### Vaccination data

To consider the precise impact of the nationwide COVID-19 vaccination on SARS-CoV-2 transmissions in Korea, information on COVID-19 vaccines administered between 6 March and 8 September 2021, was collected from non-publicly available KDCA reports (“vaccination data”). The vaccination data included the weekly number of administered doses by type (AZD1222, JNJ-78436735, BNT162b2, and mRNA-1273) and order (first and second dose) of the COVID-19 vaccine, along with the age group of vaccinated individuals. In addition, the data also includes the expected number of vaccinated individuals from 9 September to 31 December 2021, in accordance with the currently planned vaccine roll-out strategy and COVID-19 vaccine reservation data in Korea. As this study aimed to reconstruct the transmission dynamics of SARS-CoV-2 at the national level, spatial variants were not taken into consideration in all data (i.e., incidence, variant, and vaccination data).

### Model formulation and parameterization

A mathematical model incorporating transmission dynamics of SARS-CoV-2 infections by age group and by variant was developed in two steps: reconstruction of the age-stratified next-generation matrix and estimation of the relative transmissibility by variant. First, to reconstruct the next-generation matrix by fitting the age-structured model with the incidence data. Second, based on the estimated next-generation matrix, we assessed the relative transmissibility of each SARS-CoV-2 variant by fitting the expected number of cases to the reported case counts using the multivariate renewal process.

#### Reconstruction of the next-generation matrix

A discrete-time deterministic compartment model structured by age group, considering the age-stratified transmission dynamics of COVID-19 was devised to calibrate parameters and serve as a baseline model (Fig. S2; see Additional file). First, we fitted the baseline model to the incidence data during 16–27 November 2020 to estimate the relative susceptibility to SARS-CoV-2 infection in each of the four age groups, given the contact pattern between age groups in Korea (Fig. S3; see Additional file) [14]. The timeframe was intentionally determined to ensure that the corresponding data fully reflects the transmission dynamics of the wild-type variant under the minimum level of countermeasures (e.g., only retaining personal protective behaviors; 7–18 November 2020) [15] with the consideration of the empirically observed time delay of 9 days from infection to reporting. Based on the estimates, we reconstructed the next-generation matrix, accounting for the age-structured transmission dynamics of the wild-type SARS-CoV-2 variant in Korea, and derived the reproduction number (*R*) from its leading eigenvalue (Table S1; See Additional file 3–1 & 3–2).

#### Estimation of relative transmissibility by SARS-CoV-2 variant

We constructed the COVID-19 incidence by variant and by age group using the incidence and variant data from 22 June to 9 July 2021, assuming that the weekly proportion of variants remains constant throughout the week. To estimate the relative transmissibility by variant, we applied a multivariate renewal process representing the expected number of new SARS-CoV-2 infections by variant and by age group. Changes in the population susceptibility following a nationwide vaccination, along with a decline in the vaccine effectiveness as the proportion of Delta variant increased over time in Korea, were also integrated into the multivariate renewal process (See Additional file 3–3). Then, we fitted it with the constructed data and estimated (i) the reduction in transmissibility due to the enhanced social distancing countermeasures (compared to Level 1) and (ii) the relative transmissibility of new SARS-CoV-2 variants using the wild-type as a reference. The timeframe was determined based on the first day of the week (week 24 in 2021) when major Delta variant transmissions were first reported in Korea [16] and the date when the KDCA announced the implementation of Level 4 social distancing plan, to consider potential behavioral changes following the announcement (e.g., seeking to take a PCR test or reducing non-essential outings) [17]. All utilized parameters above (i.e., the relative susceptibility by age group, reduction in transmissibility due to the enhanced social distancing countermeasures, and relative transmissibility by SARS-CoV-2 variant) were jointly estimated using maximum likelihood estimation, and 95% confidence intervals (CIs) of each parameter were calculated from 5000 samples using a Laplace approximate normal distribution.

### Numerical simulations

Based on the proposed model incorporating the age- and variant-stratified SARS-CoV-2 transmission dynamics and the nationwide vaccination in Korea, we performed numerical simulations to project (i) the number of newly reported COVID-19 cases, (ii) the prevalence of severe cases, and (iii) the cumulative COVID-19 deaths. First, to evaluate the effect of Level 4 social distancing countermeasures (implemented from 12 July 2021), we examined the epidemiological outcomes related to differing levels of its impacts on the SARS-CoV-2 transmissibility (ranging from 30 to 40%). In addition, to determine an optimal strategy for controlling the current COVID-19 epidemic with the minimum level of countermeasures (Level 1), we compared the effect of different strategies with variations in the time for degrading NPIs and the number of administered vaccines, by comparing four different epidemiological outcomes: (i) the presence of resurgence after lifting interventions, (ii) the cumulative incidence after lifting interventions, (iii) the peak prevalence of severe cases after lifting interventions, and (iv) the cumulative number of deaths. In detail, the time for degrading NPIs (to Level 1 social distancing) varied with a range of 4 October–1 November 2021 (referred to as “Scenario 1–5” with a 7-days interval). The total number of administered vaccines from 4 October 2021 was varied from 5 to 20% increase (see Additional file 4–3).

To identify an optimal exit strategy by lifting all countermeasures, the proposed model was also used to assess the optimal duration of gradual relaxation (ranging from 2 to 6 months) enabling to minimize the disease burden until effective COVID-19 treatments become widely available. Given that the reported basic reproduction number of the Delta variant is near 5 [18] and the 3 T strategy would be maintained even after all interventions were lifted, the *R* of the Delta variant was modelled to increase from 1 November 2021, until it reaches to 3.5 (*R*_*max*_) following a logistic growth function. Furthermore, to address the uncertainty in the value of *R*_*max*_, possible COVID-19 waves were projected by varying the *R*_*max*_ between 3.0 and 3.5 (3.0, 3.25, and 3.5), while the duration of gradual relaxation was fixed at 2 months (from 1 November through 31 December 2021). All analyses were conducted using *R* statistical software. Full details of the parameter choices and a detailed model description can be found in the Additional file.

## Results

Fig. 1 demonstrates the age-stratified epidemic curve of COVID-19 (Fig. 1A) and the proportion of each SARS-CoV-2 variant among the tested samples by age group in Korea (Fig. 1B–E). Compared to the pre-nationwide vaccination period (before April 2021), a substantial decrease in the proportion of individuals aged over 60 years (who were prioritized for the COVID-19 vaccination) was observed, while 20–59 years became the dominant age group for confirmed COVID-19 cases. In addition, since the major transmission of the Delta variant began to be reported from week 24, the Delta variant rapidly outperformed existing SARS-CoV-2 variants and completely replaced them in week 35 across all age groups.

Table S2 (see Additional file) shows the quantified next-generation matrix of wild-type SARS-CoV-2 variant in Korea, based on the estimated age-stratified relative susceptibility and contact pattern by age groups. The reproduction number (*R*) of the wild-type variant under Level 1 countermeasures was estimated as 1.49 (95% CIs: 1.47–1.50). The modelled COVID-19 incidence based on the reconstructed next-generation matrix was mostly in line with the overall trend of observed incidence (the incidence data) in all age groups, as shown in Fig. S4 (see Additional file). In addition, Table S3 (see Additional file) demonstrates the relative transmissibility of SARS-CoV-2 variants compared with the wild-type. Given the transmissibility of the wild-type variant as a reference (set as 1), the highest estimate observed for the Delta variant was 1.60 (95% CIs: 1.54–1.67), implying that the transmissibility of the Delta variant is 1.6 times higher than that of the wild-type variant. The relative transmissibility of the Alpha variant and the other variants were estimated as 0.87 (95% CIs: 0.82–0.91) and 0.29 (95% CIs: 0.11–0.47), respectively. The inferred incidences of SARS-CoV-2 infections by variant and by age group were consistent with the overall trend of reported case counts (Fig. S5) (see Additional file), indicating that the set of estimates enabled the accurate capture of the age- and variant-stratified transmission dynamics of SARS-CoV-2 infections in Korea.

**Fig. 2:**
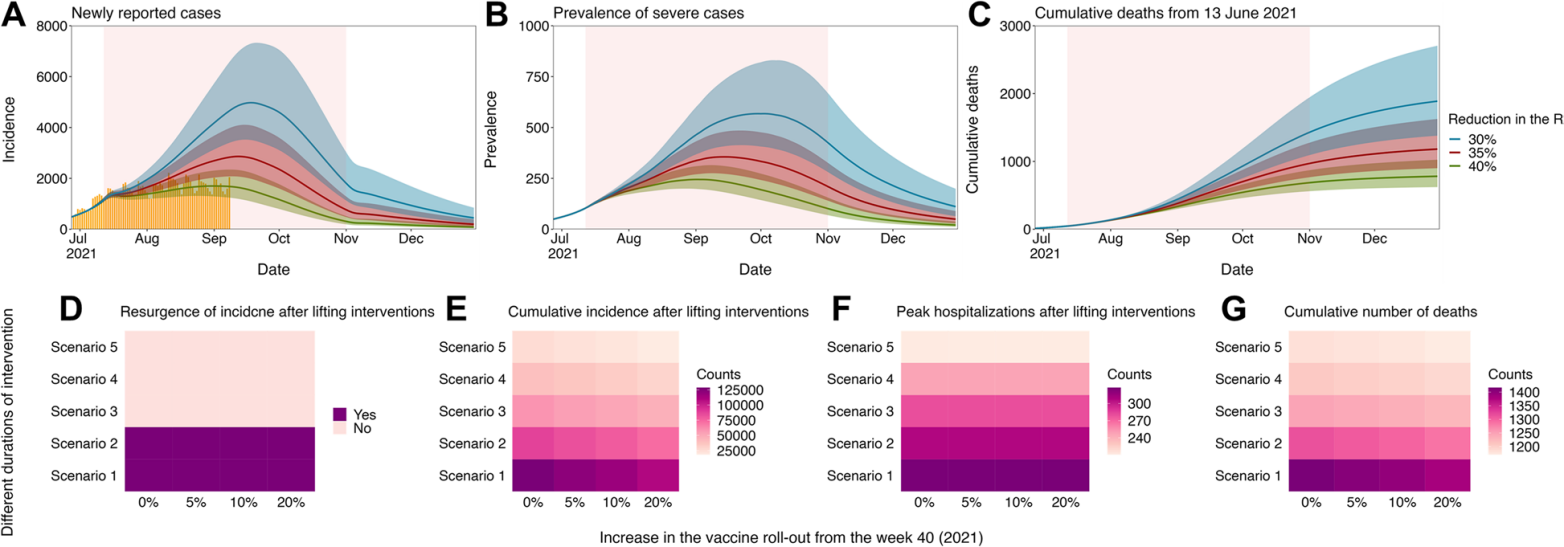
Projected waves of SARS-CoV-2 infections in the Republic of Korea. **A–C** Projected waves of SARS-CoV-2 infection under the current vaccine roll-out plan with the social distancing countermeasures downgraded from Level 4 to Level 1 from 1 November 2021: **A** the number of newly infected cases, **B** the prevalence of severe cases, and **C** the cumulative number of COVID-19 deaths from the start day of simulation (13 June 2021). In each figure, blue, red, and green lines and shaded areas indicate projected outcomes and their 95% confidence intervals given the reduction in SARS-CoV-2 transmissions by 30, 35, and 40%, respectively, due to the implementation of Level 4 social distancing. The pink shaded area shows the period during which Level 4 social distancing countermeasures were implemented. **D–G** Comparison of epidemiological outcomes by different timings of downgrading the social distancing countermeasures to Level 1 (4 October–1 November 2021 with a 7-days interval^*^) and different levels of increase in the vaccine roll-out from week 40 in 2021 (5–20%). The impacts of possible COVID-19 response stratifications were compared using four outcomes: **D** Presence of resurgence after lifting interventions, **E** cumulative incidence after lifting interventions, **F** peak prevalence of severe cases after lifting interventions, and **G** cumulative number of deaths. ^*^Scenario 1: October 4; Scenario 2: October 11; Scenario 3: October 18; Scenario 4: October 25; and Scenario 5: November 1

The projected transmission dynamics of COVID-19, assuming the currently planned nationwide vaccination program, are shown in Fig. 2A–C, along with the quantified impacts of possible COVID-19 response strategies (i.e., different timings of the Level 1 social distancing and numbers of administered vaccines from 4 October 2021) on the epidemiological outcomes (Fig. 2D–G). The comparison between the projected incidence and reported case counts shows that Level 4 social distancing measures successfully reduced the transmissibility of all SARS-CoV-2 variants by more than 40% (Fig. 2A). Given the estimated parameters, the projected epidemiological outcomes prior to the implementation of Level 4 social distancing measures (12 July 2021) were well aligned with the overall trend of empirically obtained values from the time series of COVID-19 data in Korea.

Our projection also suggests that tapering the level of social distancing to the minimum level (Level 1) from November 2021 can keep suppressing the resurgence of disease incidence unless the current vaccine roll-out plan is not well secured or new SARS-CoV-2 variants with higher transmissibility emerge in the future. In addition, despite increasing vaccine administration by 20% from October 2021, sustaining the maximum level (Level 4) of social distancing beyond 18 October 2021 is required to keep the epidemic under control without any additional resurgences (Fig. 2D). It implies the importance of maintaining NPIs until a provisional herd immunity (i.e., reaching a certain vaccination coverage that enables a decline in the spread of the disease given the transmissibility of SARS-CoV-2) is achieved by the mass vaccination.

**Fig. 3:**
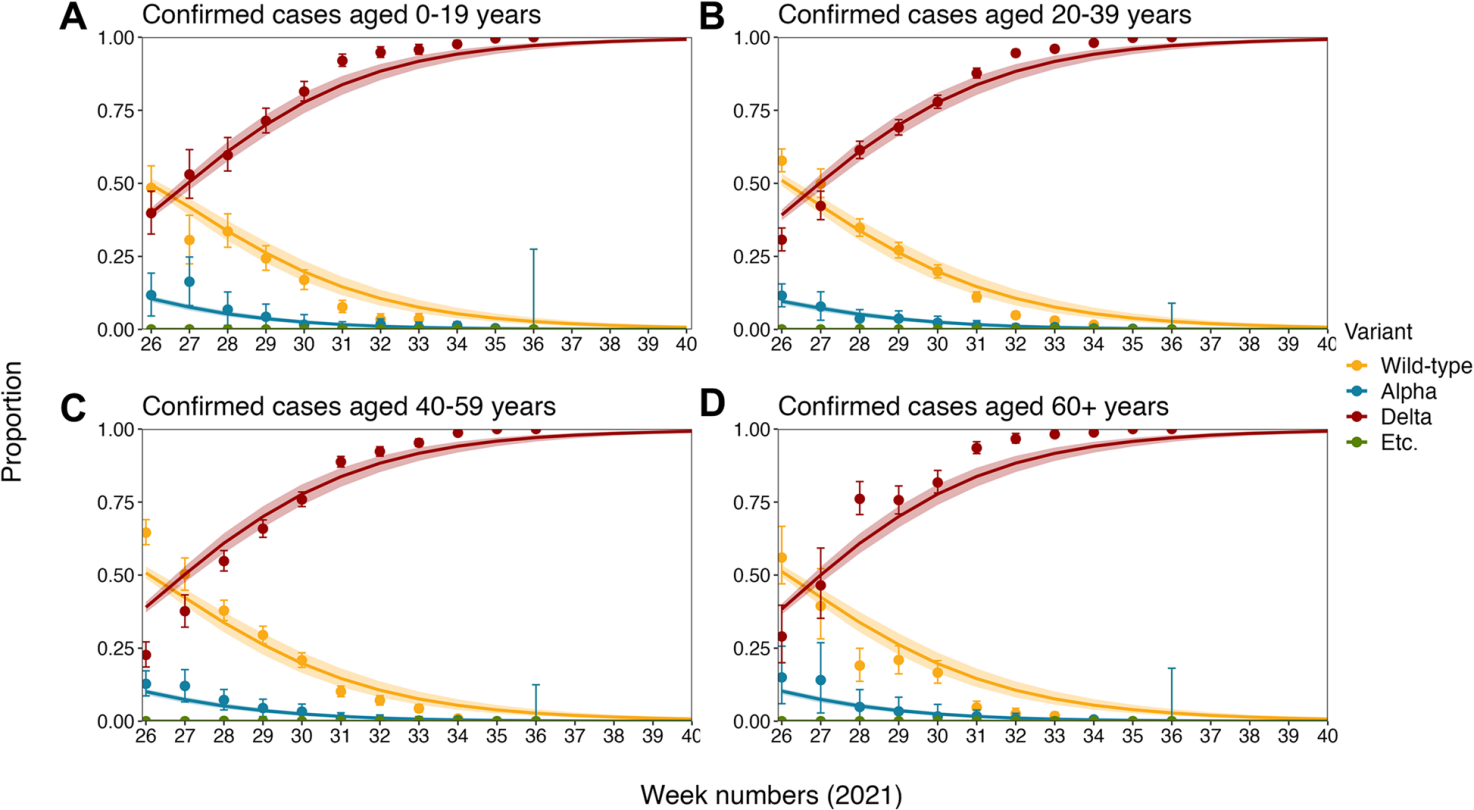
Comparison of observed and projected proportion of SARS-CoV-2 variants by age group in the Republic of Korea. Comparison between the observed and projected proportion of SARS-CoV-2 variants in the Republic of Korea by four age groups: **A** 0–19, **B** 20–39, **C** 30–49, and **D** those aged 60 and over. Points and error bars represent the observed proportion of each SARS-CoV-2 variant, i.e., Wild-type, Alpha variant, Delta variant, and the other variants (Etc.), and their 95% confidence intervals from non-publicly available reports from the Korea Disease Control and Prevention Agency. Lines and shaded areas are projected results with 95% confidence intervals using the proposed model, given that the transmissibility of all SARS-CoV-2 variants has dropped by 40% due to Level 4 social distancing

Given that the transmissibility of all SARS-CoV-2 variants has dropped by 40% due to Level 4 social distancing, the projected and observed proportion of each SARS-CoV-2 variant by age group were compared to conduct cross-validation (Fig. 3). The projected values and their 95% CIs traced the overall trend of the observed proportion of each SARS-CoV-2 variant in all age groups (the variant data), demonstrating the validity of our projection. In addition, the result endorses the potential for dominance of the Delta variant, suggesting that it will entirely replace all existing SARS-CoV-2 variants across all age groups from September 2021.

**Fig. 4:**
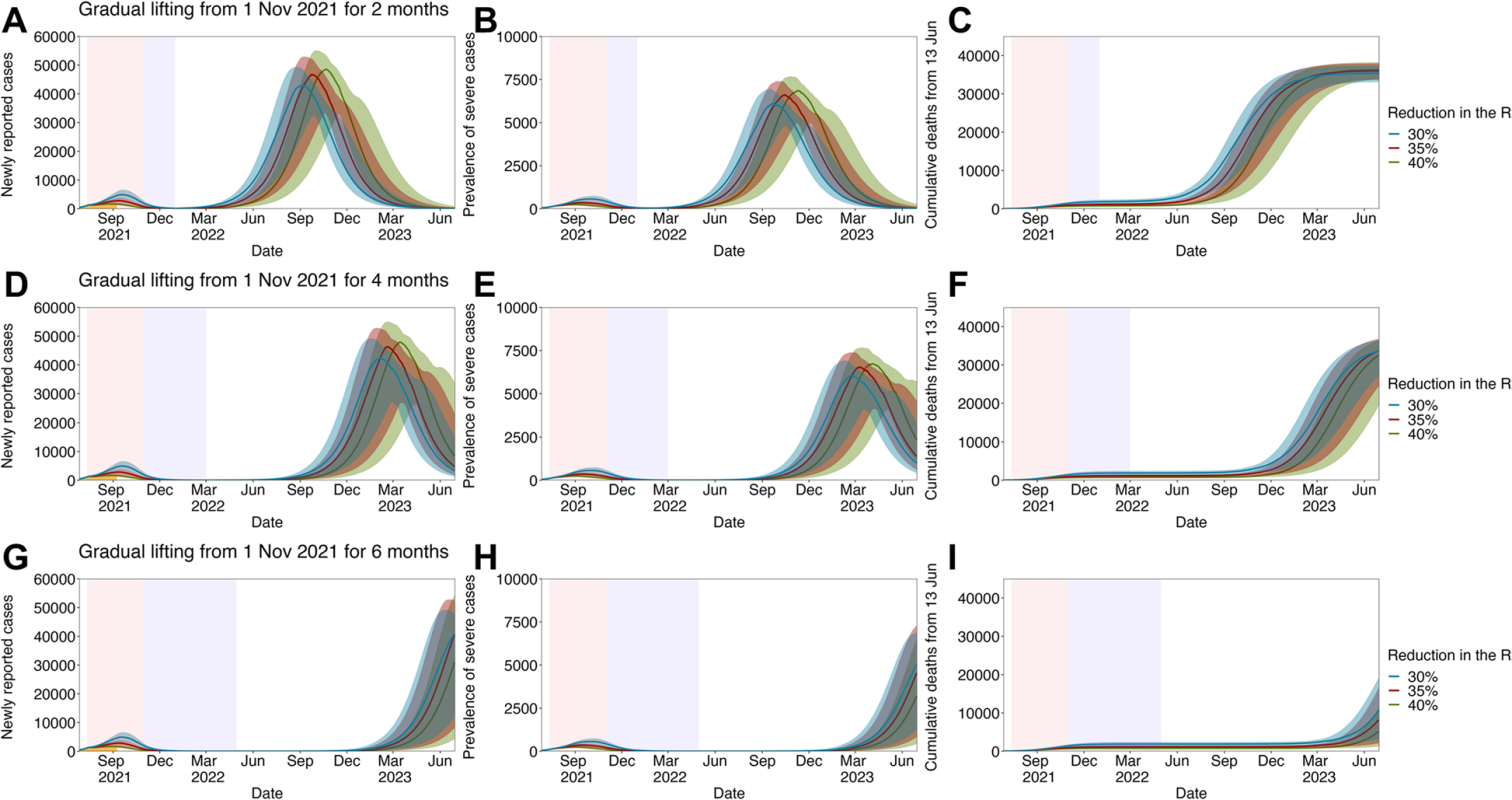
Long-term projection of SARS-CoV-2 infections in the Republic of Korea by varying the duration for the gradual relaxation of interventions. Projected waves of SARS-CoV-2 infection given the full lifting of interventions with three epidemiological outcomes: the newly reported cases, the prevalence of severe cases, and the cumulative number of deaths from 13 June 2021. In each projection, the reproduction number of the Delta variant was assumed to follow the logistic growth function with the fixed maximum value (*R*_*max*_ =3.5) and the varied duration for the gradual relaxation of interventions: for **A–C** 2 months, **D–F** 4 months, and **G–I** 6 months. In each figure, blue, red, and green lines and shaded areas indicate projected outcomes and their 95% confidence intervals given the reduction in SARS-CoV-2 transmissions by 30, 35, and 40%, respectively, due to the implementation of Level 4 social distancing. In addition, the pink shaded area shows the period in which Level 4 social distancing countermeasures were implemented, whereas the purple shaded area represents the period for gradual relaxation

**Fig. 5:**
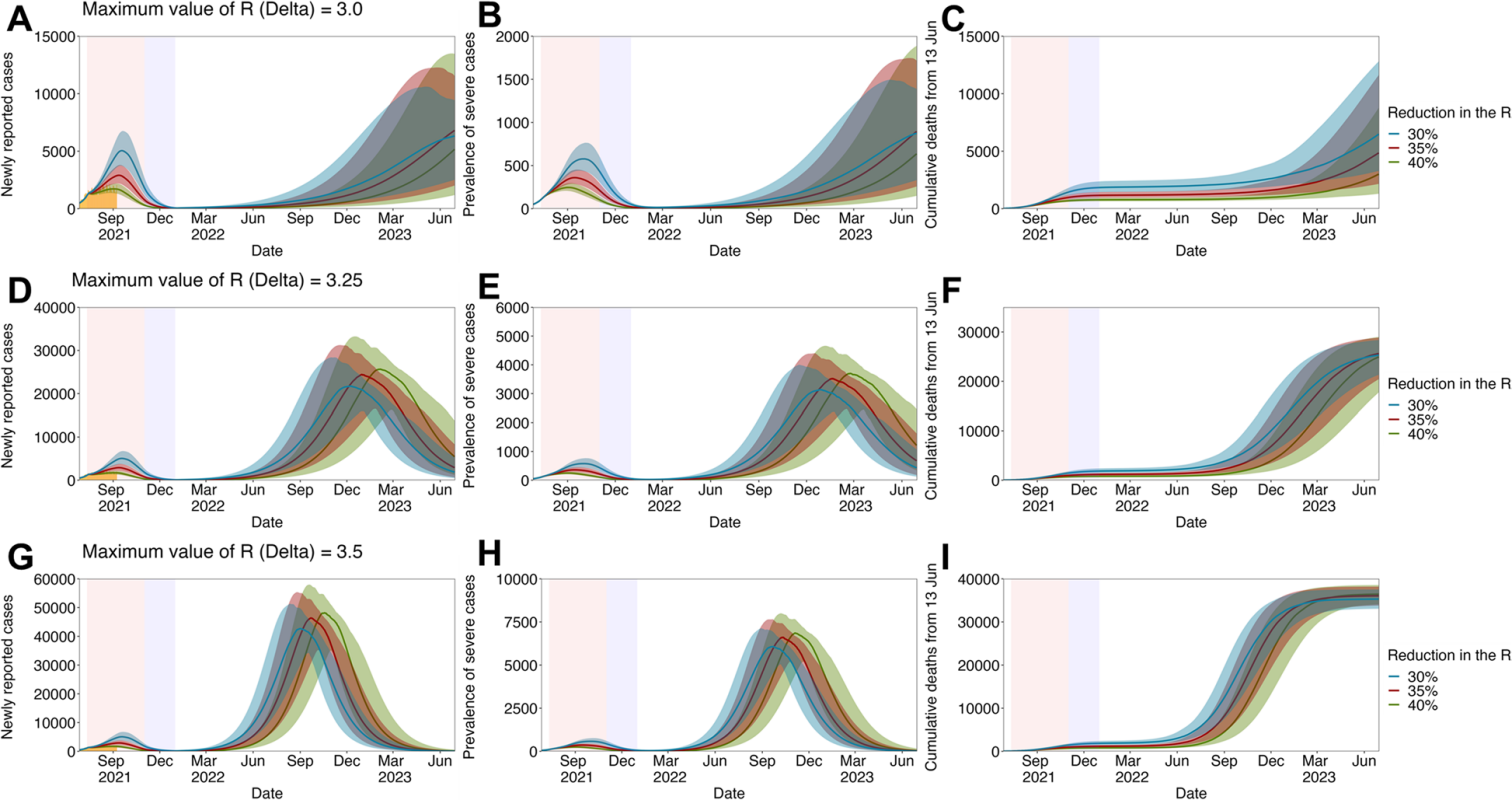
Long-term projection of SARS-CoV-2 in the Republic of Korea by varying the maximum reproduction number of the Delta variant (*R*_*max*_). Projected waves of SARS-CoV-2 infection given the full lifting of interventions with three epidemiological outcomes: the newly reported cases, the prevalence of severe cases, and the cumulative number of deaths from 13 June 2021. In each projection, the reproduction number of the Delta variant was assumed to follow the logistic growth function with the 2 months of gradual increase and the varied maximum value: **A–C** *R*_*max*_ =3.0, **D–F** *R*_*max*_ =3.25, and **G–I** *R*_*max*_ =3.5. In each figure, blue, red, and green lines and shaded areas indicate projected outcomes and their 95% confidence intervals given the reduction in transmissibility of SARS-CoV-2 variants by 30, 35, and 40%, respectively, due to the implementation of Level 4 social distancing. In addition, the pink shaded area shows the period in which Level 4 social distancing countermeasures were implemented, whereas the purple shaded area represents the period for gradual relaxation (fixed at 2 months)

Fig. 4 shows the quantified impacts of exit strategies with the full lifting of all countermeasures followed by different durations of gradual relaxation from November 2021. Assuming that the maximum *R* of the Delta variant (*R*_*max*_) is 3.5, our model suggested that gradual easing for more than 4 months is sufficient to withstand the prevalence of severe COVID-19 cases within the existing intensive care unit (ICU) capacity in Korea (800–1000 units) until the end of 2022, enabling the control of the current COVID-19 epidemic before prospective treatments of COVID-19 are widely available. However, as shown in Fig. 5, under the setting when countermeasures are partially maintained and the *R*_*max*_ is sustained below the value of 3, the exit strategy with only 2 months of gradual easing can allow socio-economic activities to be resumed while minimizing the burden of the disease until late 2022.

## Discussion

In the present study, we demonstrated a model-informed approach to project short- and long-term trends of the COVID-19 epidemic, using accessible epidemiological data in Korea. In particular, the proposed model could estimate the relative transmissibility of new SARS-CoV-2 variants and project the transmission dynamics of each variant based on the estimates. By applying the very limited data (only the initial phase of the Delta variant transmissions) into our model framework, our projection was well aligned with the empirically observed time series of COVID-19 incidence in Korea. Additionally, the proposed model quantitatively assessed the impact of prospective exit strategies with different timings and levels of restrictions and provided insight into an optimal exit strategy that allows socio-economic activities to resume, while minimizing the burden of ongoing transmission of SARS-CoV-2. However, it should be noted that the projected final size of the epidemic may differ from real world, since various NPIs are highly likely implemented to suppress such a rapid rise of incidences and hospitalizations in practice.

In our study, while the transmissibility of the Delta variant was estimated to be the highest, that of the Alpha variant was estimated to be lower than that of the wild-type variant, which is not consistent with conventional knowledge [19]. However, in November 2020 the dominant SARS-CoV-2 variant in Korea (which is referred to as “wild-type variant” in the present study) belonged to the B.1.497 lineage [20, 21], and it is suggested to have higher transmissibility than the original Wuhan strain owing to the D614G mutation on the spike protein [22, 23]. Thus, the conventional knowledge might not be valid for the situation where the dominant SARS-CoV-2 variant has an equivalent or higher transmissibility than that of the Alpha variant. Furthermore, from mid-June 2021, the Alpha variant began to decline in frequency, while circulation of the wild-type variant was prolonged through large-size clusters in metropolitan areas (e.g., a nightclub cluster with more than 200 confirmed infections) [24]. Therefore, it is noteworthy that our estimates of relative transmissibility relying on the corresponding incidence data might underestimate the actual transmissibility of outcompeted variants. However, despite this constraint, the projected proportion of SARS-CoV-2 variants well captured the empirically observed values, and such performance of the proposed model suggests that our estimates can provide credible projections of future SARS-CoV-2 infections.

Our main finding suggests that countries that have controlled the spread of COVID-19 relatively well such as Korea, Japan, Australia, and New Zealand may face a new challenge in developing an effective COVID-19 exit strategy. Considering the negligible socio-economic impacts of stringent restrictions, it cannot be maintained for an extended period of time and the coexistence with COVID-19 seems inevitable. However, due to the relatively low number of cumulative incidences, a hasty relaxation of social distancing measures relying only on vaccination coverage could result in a considerable increase in the burden of disease in Korea [25, 26], as clearly indicated by a huge surge of incidence in our projections. Indeed, the proportion of cumulative confirmed cases in Korea, Japan, and Australia was only less than 1% of the total population as of 1 November 2021, which is much lower than the United Kingdom (12.13%) and Denmark (6.3%) [5]. This implies the consequence of complete easing of countermeasures can be much bigger in Korea, compared to countries where restrictions have already been lifted such as the United Kingdom or the United States.

The next key question for those countries is how to keep the epidemic under control after lifting the restrictions on physical contact, and our projections suggest a gradual relaxation of NPIs for more than 4 months can withstand the prevalence of COVID-19 hospitalizations under the existing ICU capacity in Korea until the end of 2022. With successful results in clinical trials of anti-viral medications [27], keeping the disease contained for as long as possible can be an effective way to control the current epidemic, while the national government also needs to prepare a distinct COVID-19 response strategy including a prompt administration of those treatments and resolving a stagnation in vaccine uptake. In addition, a temporary introduction of the “circuit breaker” measure (i.e., a tight set of restrictions for a limited amount of time) can be considered to relieve unsustainable pressure on the healthcare settings [28]. However, given the uncertainty surrounding the basic reproduction number of the Delta variant and the emergence of more transmissible SARS-CoV-2 variants, the risk assessment should be conducted on a regular basis based on the existing health care capabilities and scenario-based projection of transmission dynamics.

Three main limitations of this study should be also discussed. First, when we constructed the next-generation matrix, the overall profile of age-specific susceptibility was assumed to be identical to that of the wild-type variant, while the degree of age-specific susceptibility was adjusted following the estimated relative transmissibility, in order to alter the reproduction number of each variant. However, if the age-specific transmission profile differs by variant, the projected transmission dynamics of COVID-19 and its epidemiological outcomes, especially the prevalence of severe cases and the cumulative number of deaths, can be affected. Second, our model did not adequately account for the additional features of new SARS-CoV-2 variants (e.g., cross-reactive immunity across variants or immune escape). If new SARS-CoV-2 variants can evade natural and vaccinal immunity more efficiently, the projected number of incidences and deaths can be altered due to their effects on the vaccine effectiveness. Nonetheless, given the currently available information, the overall impacts of possible exit strategies could be evaluated through the proposed model. Lastly, although various aspects of COVID-19 vaccines were addressed, our model still relies on the assumption that susceptible individuals will be promptly and completely protected against SARS-CoV-2 infection given the certain vaccine effectiveness. It might underestimate simulated outcomes due to the time delay from vaccination to immune protection [29] and reported waning immunity as a function of time [30]. However, the waning immunity will have little impact on our projected results, given the relatively late vaccine roll-out in Korea, as well as the planned adoption of a booster vaccination targeting high-risk individuals who were prioritized in the initial vaccination.

## Conclusion

The present study suggests that an exit strategy with the gradual lifting of restrictions is essential to sustain the suppression of SARS-CoV-2 transmissions considering the transmission dynamics of new SARS-CoV-2 variants. Despite the uncertainties surrounding the new variants, our simulations provide evidence-based guidance for an exit strategy that returns society to normalcy, especially in countries where the spread of COVID-19 has been well controlled.

## Supplementary Information

**Additional file 1.**

## Data Availability

The datasets generated during and/or analyzed during the current study are not publicly available due to the Korea Disease Control and Prevention Agency’s policy. But are available from the corresponding author on reasonable request. All requests will be reviewed by Korea Disease Control and Prevention Agency.

## Abbreviations

CIs: Confidence intervals
COVID-19: Coronavirus disease 2019
ICU: Intensive care unit
KDCA: Korea Disease Control and Prevention Agency
NPIs: Non-pharmaceutical interventions
SARS-CoV-2: Severe acute respiratory syndrome coronavirus 2
