# Supplementary material for: Model-informed COVID-19 exit strategy with projections of SARS-CoV-2 infections generated by variants in the Republic of Korea
Source: BMC Public Health. 2022 Nov 17;22:2098. doi: 10.1186/s12889-022-14576-w (PMC9668700; doi:10.1186/s12889-022-14576-w)
Supplement: Supplementary file 1 — Additional file 1. [file 12889_2022_14576_MOESM1_ESM.docx]

**[Additional file]**

**Model-informed COVID-19 exit strategy with projections of SARS-CoV-2 infections generated by variants in the Republic of Korea**

**Authors:** Sung-mok Jung^1,2^, Kyungmin Huh^3^, Munkhzul Radnaabaatar^4^, Jaehun Jung^4,5*^

**Affiliations:**

^1^Kyoto University School of Public Health, Yoshidakonoe cho, Sakyo ku, Kyoto city, 6068501, Japan

^2^Graduate School of Medicine, Hokkaido University, Kita 15 Jo Nishi 7 Chome, Kita-ku, Sapporo-shi, Hokkaido 060-8638, Japan

^3^Division of Infectious Diseases, Department of Medicine, Samsung Medical Center, Sungkyunkwan University School of Medicine, Seoul, 06351, Korea

^4^Artificial Intelligence and Big-Data Convergence Center, Gil Medical Center, Gachon University College of Medicine, Incheon, 21565, Korea

^5^Department of Preventive Medicine, Gachon University College of Medicine, Incheon, 21565, Korea

^*^**Address for Correspondence**:

Jaehun Jung, MD, PhD

Department of Preventive Medicine, Gachon University College of Medicine,

38-13, Dokjeom-ro 3, Incheon, 21565, Korea.

Tel.: +82 10 6359 3201;

Fax: +82 32 458 2608

E-mail: eastside1st@gmail.com; ORCID ID: <https://orcid.org/0000-0002-4856-3668>

**Contents**

1. **Implemented social distancing countermeasures in the Republic of Korea**
2. **Contact matrix in the Republic of Korea**
3. **Baseline model**
4. **Numerical simulations for projecting waves of SARS-CoV-2 infections**
5. **Supplementary Information Figures**
6. **Supplementary Information Tables**
7. **References**

**1. Implemented social distancing countermeasures in the Republic of Korea**

To control the ongoing coronavirus disease 2019 (COVID-19) epidemic, a social distancing countermeasure composed of four different levels has been implemented in the Republic of Korea (hereafter, “Korea”) from 7 November 2021. The level of social distancing has been determined in accordance with the weekly number of newly reported COVID-19 cases as the main indicator, along with seven sub-indicators (i.e., the weekly incidence of individuals aged 60 years and over, the bed capacity for severe COVID-19 cases, the capacity for epidemiological investigation, the effective reproduction number, the number of clusters, the proportion of cases under investigation, and the fraction of newly confirmed cases during the 14-days quarantine)^1^. The criteria for each level and detailed restrictions on physical contact by level were shown in Table S1, and Figure S1 demonstrates the level of social distancing countermeasures implemented in the metropolitan area (i.e., Seoul, Gyeonggi, and Incheon) of Korea, where the majority of confirmed COVID-19 cases (68%) has been reported.

**2. Contact matrix in the Republic of Korea**

An available age-stratified contact matrix ($m$) of Korea was constructed based on the 16 age groups^2^. Therefore, we modify the $m$ to the required format of the matrix ($M$) with four age groups employed in the present study (i.e., under 20, 20–39, 40–59, and older than 60 years). The methodology for this transformation was borrowed from the previous publication^3^. First, the total number of contacts between age groups *i* and *j* was obtained by making the symmetric matrix ($M_{ij}^{sym}$) as follow:

$$M_{ij}^{sym}=\frac{m_{ij}N_{i}+m_{ji}N_{j}}{2}, (1)$$

where $m_{i,j}$ stands the element of the published contact matrix, $m$ and $N_{i}$ is the population size of 16 age groups. Then, through summing up the corresponding elements of $M_{ij}^{sym}$ and dividing row-wise of the matrix by the population size of each age group, the rescaled four-by-four contact matrix ($M_{ij}$) was generated (Figure S2).

**3. Baseline model**

*3-1. Structure of the baseline model*

A discrete-time deterministic compartment model structured by age group by severe acute respiratory syndrome coronavirus 2 (SARS-CoV-2) variant (i.e., categorized into four types in our study: the wild-type, Alpha variant, Delta variant, and the other variants) was used for the baseline model. The baseline model was composed of six states: susceptible ($S$), exposed ($E$), infectious ($I$), hospitalized ($H$) (i.e., administered in the intensive care unit (ICU), indicating severe COVID-19 cases), recovered ($R$), and deceased ($D$). A schematic diagram for the baseline model is shown in Figure S3. First, susceptible individuals who contact an infectious individual with variant $v$ transits to exposed states ($E$), following the force of infection, $\lambda_{i,v}$. The $\lambda_{i,v}$ for a susceptible individual in age group $i$was modeled as follow^4^:

$$\lambda_{i,v}={hk_{v}u}_{i} \sum_{j} M_{ij} \frac{I_{j,v}}{N_{j}-{(H_{j}+D}_{j})}, \left( 2 \right)$$

where $u_{i}$ stands the probability of a successful transmission given a single contact with an infectious individual and $M_{ij}$ is the rescaled contact matrix defined above. $h$ represents the reduction in transmissibility of SARS-CoV-2 variants following the enhanced social distancing countermeasures (compared to Level 1 social distancing; details are in Section *3-3*), whereas $k_{v}$ indicates the relative transmissibility of variant $v$ using that of the wild-type variant as a reference. $N_{j}$ is the total population size in age group $j$, corresponding with the sum of individuals in age group $j$ in all six states (i.e., $N_{j}=S_{j}+E_{j}+I_{j}+{H_{j}+R}_{j}+D_{j}$). After contracting the disease, exposed individuals become to be infectious after a latent period ($1/\epsilon$) and infectious individuals move to either recovered states ($R$) or hospitalized states ($H$), following the empirically observed age-specific risk of severity (${SR}_{i}$)^5^, and infectious period ($1/\sigma$). In addition, hospitalized individuals (i.e., severe COVID-19 case) transition to either recovered ($R$) or deceased ($D$) states with the age-specific fatality risk among hospitalized cases (${FR}_{i}$) and duration of hospitalization ($1/\omega$). The age-specific ${FR}_{i}$ was calculated using the age-specific infection fatality risk (${IFR}_{i}$) of COVID-19^6^ and empirically observed ${SR}_{i}$ as follow:

$${FR}_{i}=\frac{{IFR}_{i}}{SR_{i}}. (3)$$

*3-2. Reconstruction of the next generation matrix of wild-type SARS-CoV-2 variant*

To estimate the relative susceptibility in age group $i$, $u_{i}$, the baseline model was fitted to the incidence data between 16–27 November 2020. We were motivated to analyze the data from this timeframe, because there was no report of new SARS-CoV-2 variants in Korea before 28 December 2020^7^, implying that all reported cases were of the wild-type variant ($k_{v}$ was set as 1). Furthermore, since the minimum level of social distancing countermeasure (Level 1) was implemented during the corresponding period, the incidence data was presumed to be unadulterated by stringent non-pharmaceutical interventions. The expected number of newly reported case with the wild-type variant on day $t$, ${E(Reported}_{i}(t))$, was modeled as follow:

$${E(Reported}_{i}(t))=\int_{\tau=1}^{t-1} {\beta SI}_{i}\left( t-\tau\right)g\left( \tau\right), \left( 4 \right)$$

where ${\beta SI}_{i}(t)$ is the number of newly infected cases with the wild-type SARS-CoV-2 variant in age group $i$ at time $t$ (i.e., the number of individuals move from $S$ to $E$ state through contact with infectious individuals on day $t$). $g(\tau)$ is the probability mass function of time delay from infection to reporting, calculated from the convolution between the distribution of incubation period^8^ and empirically observed time delay from the illness onset to reporting in Korea by fitting with a Weibull distribution (mean=3.6 days, SD=2.5 days). We assumed that ${Reported}_{i}(t)$ follows a Poisson distribution and the age-stratified relative susceptibilities ($u_{i}$), governing ${\beta SI}_{i}\left( t-\tau\right)g(\tau)$, were estimated using maximum likelihood estimation. The comparison of observed and modeled number of COVID-19 incidence by age group is shown in Figure S4.

Given the estimated $u_{i}$, we devise the structure of next-generation matrix ($K$) given by the following matrix product:

$$K=D_{u}MD_{\sigma}, \left( 5 \right)$$

where the $D_{u}$ and $D_{\sigma}$ are diagonal matrices with diagonal entries $u_{i}$ and $\sigma$, respectively. The infectious period of COVID-19 ($\sigma$) was assumed to be identical across all age groups as of 5 days^9^. The reproduction number ($R$) was derived from its leading eigenvalue of $K$. The median, the lower and upper boundaries of the 95% confidence intervals were obtained from 5,000 samples from a Laplace-approximate normal distribution (Table S2).

*3-3. Estimation of relative transmissibility by SARS-CoV-2 variant*

Assuming that the weekly proportion of SARS-CoV-2 variant $v$ in age group $i$ ($p_{i,a}$) is constant over the week, the number of new SARS-CoV-2 infections by variant $v$ in age group $i$ on day $t$, $C_{v,i}\left( t \right)$, can be modelled as:

$$C_{v,i}\left( t \right)=p_{v,i}\left( t \right)I_{i}\left( t \right), (6)$$

where $I_{i}(t)$ is the reported case counts of COVID-19 in age group $i$ combining all variants. Then, given the $C_{v,i}\left( t \right)$, the expected number of new SARS-CoV-2 infections by variant $v$ in age group $i$ on day $t$ can be formulated using the multivariate renewal process as follow:

$$E\left( C_{v, i}\left( t \right) \right)={hk_{v}R}_{ij}\sum_{\tau=1}^{t-1} \sum_{j} C_{v, j}\left( t-\tau\right)g\left( \tau\right)(1-{vax}_{i}(t)), (7)$$

where $C_{v,j}\left( t \right)$ represents the incidence of variant $v$, in age group $j$ on day $t$. $g(\cdot)$ is the probability mass function of generation time^10^, which is assumed to be identical across all SARS-CoV-2 variants. $R_{ij}$ is the effective reproduction number of wild-type SARS-CoV-2 variant, interpretable as the average number of secondary wild-type cases in age group *i* induced by a single primary wild-type case of age group *j*, which is derived from the reconstructed next-generation matrix (Section *3-2*). Lastly, ${vax}_{i}(t)$ is the proportion of immunized individuals by the nationwide COVID-19 vaccination in age group $i$ on day $t$, considering the vaccine effectiveness against SARS-CoV-2 infections by types (AZD1222, JNJ-78436735, BNT162b2 and mRNA-127) and orders (1st and 2nd dose) of available COVID-19 vaccines, along with decreases in the vaccine effectiveness followed by the proportion of Delta variant among all tested samples. Accordingly, ${vax}_{i}\left( t \right)$ was modelled as:

$${vax}_{i}\left( t \right)=\sum_{g} \frac{\left( \begin{aligned} {dose}_{i,1,g}\left( t \right)\left( {ve}_{org,1,g}\left( 1-p_{\delta,i}\left( t \right) \right)+{ve}_{\delta,1,g}p_{\delta,i}\left( t \right) \right)+ \\ {dose}_{i,2,g}\left( t \right)\left( {ve}_{org,g,2}\left( 1-p_{\delta,i}\left( t \right) \right)+{ve}_{\delta,2,g}p_{\delta,i}\left( t \right) \right) \end{aligned} \right)}{N_{i}}, \left( 8 \right)$$

where ${dose}_{i,1,g}(t)$ denotes the number of individuals in age group $i$ who received the 1st dose of type $g$ COVID-19 vaccine on day $t$. $p_{\delta}(t)$ is the empirically observed proportion of the Delta variant among all tested samples in age group $i$ on day $t$ from the variant data. ${ve}_{org,1,g}$ is the vaccine effectiveness of the 1st dose of type $g$ vaccine against the wild-type SARS-CoV-2 infection, whereas ${ve}_{\delta,1,g}$ represents the vaccine effectiveness against the Delta variant infection. In our model, a prompt and lifelong vaccinal immunity was assumed to be acquired from the day of immunization, given the vaccine effectiveness by order and by type of COVID-19 vaccines.

We fitted the multivariate renewal process (*equation 7*) to the constructed age- and variant-stratified incidence data from 22 June–9 July 2021. The timeframe was determined based on the first day of week (week 24 in 2021) when major transmissions of the Delta variant were first reported in Korea^7^ (variant data) and the date when KDCA announced the plan for imposing a Level 4 social distancing from 12 July 2021^11^ to consider behavior changes after the announcement (e.g., seeking hospital to take a PCR test or reducing non-essential outings). All parameters (i.e., $u_{i}$, $h$, and $k_{v}$) utilized in Section *3-2* and *3-3*, were jointly estimated using the maximum likelihood estimation and 95% confidence intervals of each parameter were calculated from 5,000 samples from a Laplace approximate normal distribution. The comparison between the observed and modeled number of COVID-19 incidence by age group and by variant is shown in Figure S5 and full details of the parameter choices are shown in Table S3.

**4. Numerical simulations for projecting waves of SARS-CoV-2 infections**

*4-1. Consideration of a nationwide COVID-19 vaccination*

To precisely project transmission dynamics of COVID-19 under the current nationwide vaccination in Korea, a compartment for individuals who are fully protected against SARS-CoV-2 infection by vaccination ($V_{i}$) was also introduced in the baseline model, assuming an “all-or-nothing” vaccine. Accordingly, the number of individuals who move from $S_{i}$ state to $V_{i}$ state in age group $i$ on day $t$ is determined based on the *equation* 8, calculated using a model-informed $p_{\delta}\left( t \right)$ and the currently planned vaccine roll-out strategy in Korea, ${dose}_{i,g}(t)$. In our study, ${vax}_{i}\left( t \right)$ was also calculated taking account of the reduction in vaccine effectiveness as the Delta variant spreads. Thus, under the circumstance that administered dose of vaccines is small while the fraction of Delta variant considerably grows, we allow the model to transit individuals from $V_{i}$ state to $S_{i}$ state. Furthermore, to account for the fact that the vaccine effectiveness against hospitalization is higher than that against COVID-19 infection^12^, the risk of severity in age group $i$ (${SR}_{i}$) was modelled to linearly decrease until it reached 5% of reported values, following the proportion of vaccinated individuals (i.e., counting all vaccinated individuals regardless of whether or not they have received complete protection by vaccination) in each age group and assuming the vaccine effectiveness against hospitalization across all types of vaccine as of 95%^13^.

*4-2. evaluating the impacts of Level 4 social distancing countermeasures on the transmission dynamics of COVID-19*

To assess the impacts of Level 4 social distancing countermeasures on the transmissibility of SARS-CoV-2 variants, we applied the suggested model to project future waves of SARS-CoV-2 infections with variations in the level of its impact (ranged from 30 to 40%). Accordingly, the projected number of newly reported COVID-19 cases, considering the current vaccine roll-out plan, was compared with the observed case counts from 27 June to 8 September 2021. The prevalence of severe cases and cumulative number of COVID-19 deaths were projected, in addition to the number of newly reported cases, to assess the overall impacts on the transmission dynamics of COVID-19 in Korea.

*4-3. Assessment of possible COVID-19 response strategies under the minimum level of social distancing countermeasures*

To examine an optimal COVID-19 response strategies allowing to suppress the current COVID-19 epidemic with Level 1 social distancing, we compared epidemiological outcomes, by simulating the model from 13 June 2021 and by varying the (i) timing of degrading the social distancing countermeasures and (ii) administered dose of vaccines. In detail, to determine possible transmission dynamics of SARS-CoV-2 variants under Level 1 social distancing countermeasures with different timings of downgrading, the relative reduction in transmissibility due to the intensified interventions ($h$ in *equation* 2) was adjusted to 1 (i.e., identical transmissibility with November 2020) from the assumed lifting day in each scenario (4 October–1 November 2021 with a 7-days interval). Also, the expected number of newly vaccinated individuals from 4 October 2021 was varied to quantify its impact on the transmission dynamics, and accordingly we increased the overall amount of expected vaccine doses to be administered between 4 October and 31 December 2021 by 5–20%. In each simulation, the projection was conducted from 13 June 2021 through 31 December 2021.

*4-4. Assessment of possible exit strategies with the full lifting of all countermeasures*

It is unlikely that the stringent interventions can be maintained for an extended period of time given its considerable impacts on society (e.g., the economic, social, and psychological impacts), while a prompt resume of socioeconomic activities may lead to re-emergence of cases. Therefore, we applied the proposed model to identify an optimal duration of a gradual relaxation before lifting all countermeasures in Korea, by comparing the number of newly reported COVID-19 cases and prevalence of severe cases related to differing durations of gradual easing (ranged 2–6 months). In light of the effect of 3T strategy on the partial suppression of the spread of the Delta variant (which was suggested to be a dominant variant in Korea from late September 2021), we assumed the maximum $R$ value of the Delta variant ($R_{max}$) as of 3.5. Then, we projected future SARS-CoV-2 transmissions by modelling the $R$ of Delta variant to increase until it reaches 3.5 following a logistic growth function from 1 November 2021, while altering the point of maximum growth (i.e., the duration for the gradual relaxation of interventions). Furthermore, to account for the uncertainty in the value of $R_{max}$, possible COVID-19 waves were projected by adjusting the $R_{max}$ between 2.75 and 3.5 (2.75, 3.0, 3.25, and 3.5), with the fixed duration of gradual relaxation as 2 months (from 1 November through 31 December 2021).

**5. *Supplementary Information* Figures**

**
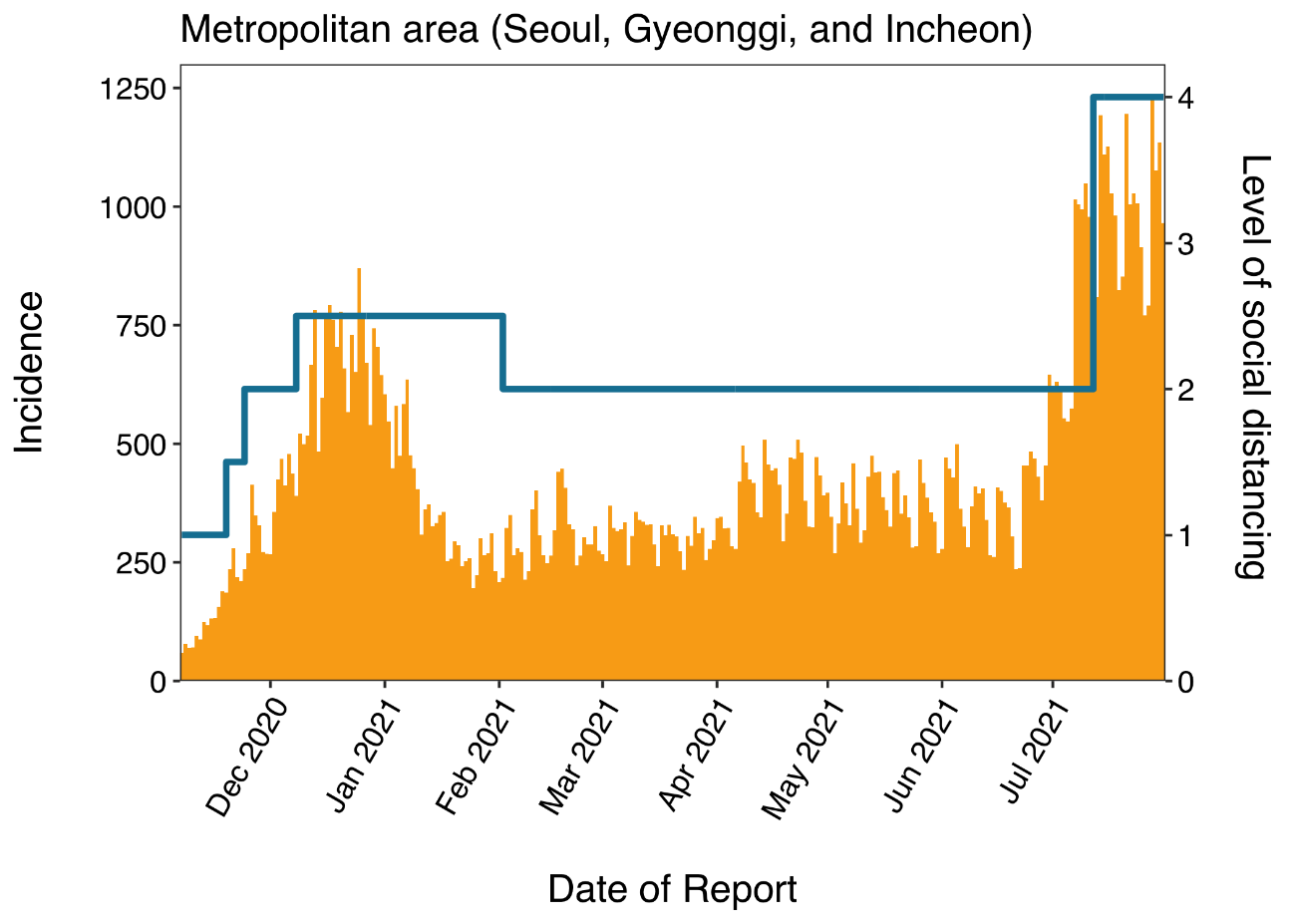
**

**Figure S1. Implemented level of social distancing countermeasures in the metropolitan area in the Republic of Korea**

Epidemic curve of COVID-19 with the implemented level of social distancing countermeasures by calendar time. Yellow bars represent the reported case counts of COVID-19 in Korea, while the blue line indicates the level of social distancing countermeasures (Level 1 through Level 4) implemented in the metropolitan area (i.e., Seoul, Gyeonggi, and Incheon) where the majority of cases have been reported.

**
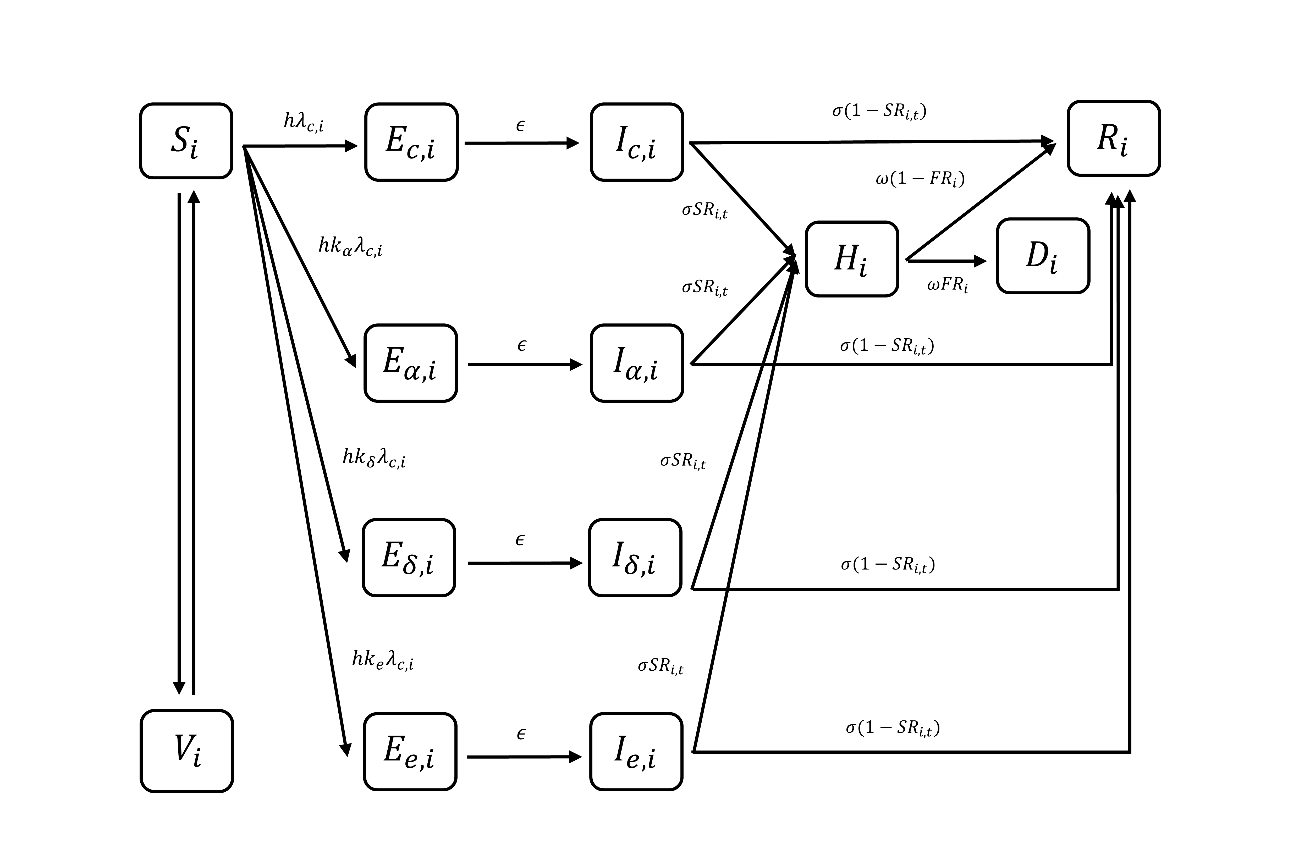
Figure S2. Schematic diagram for modeling framework**

Diagram shows a compartment model and transition rates for the baseline model incorporated with vaccination. $S$, $E$, $I$, $R$, $H$, and $D$ denote susceptible, exposed, infectious, recovered, hospitalized (i.e., severe COVID-19 cases who administers in an intensive care unit) and deceases states, respectively, and compartment $V$ indicates those who are entirely protected from SARS-CoV-2 infections by vaccination. All compartments are stratified by age group $i$.

**
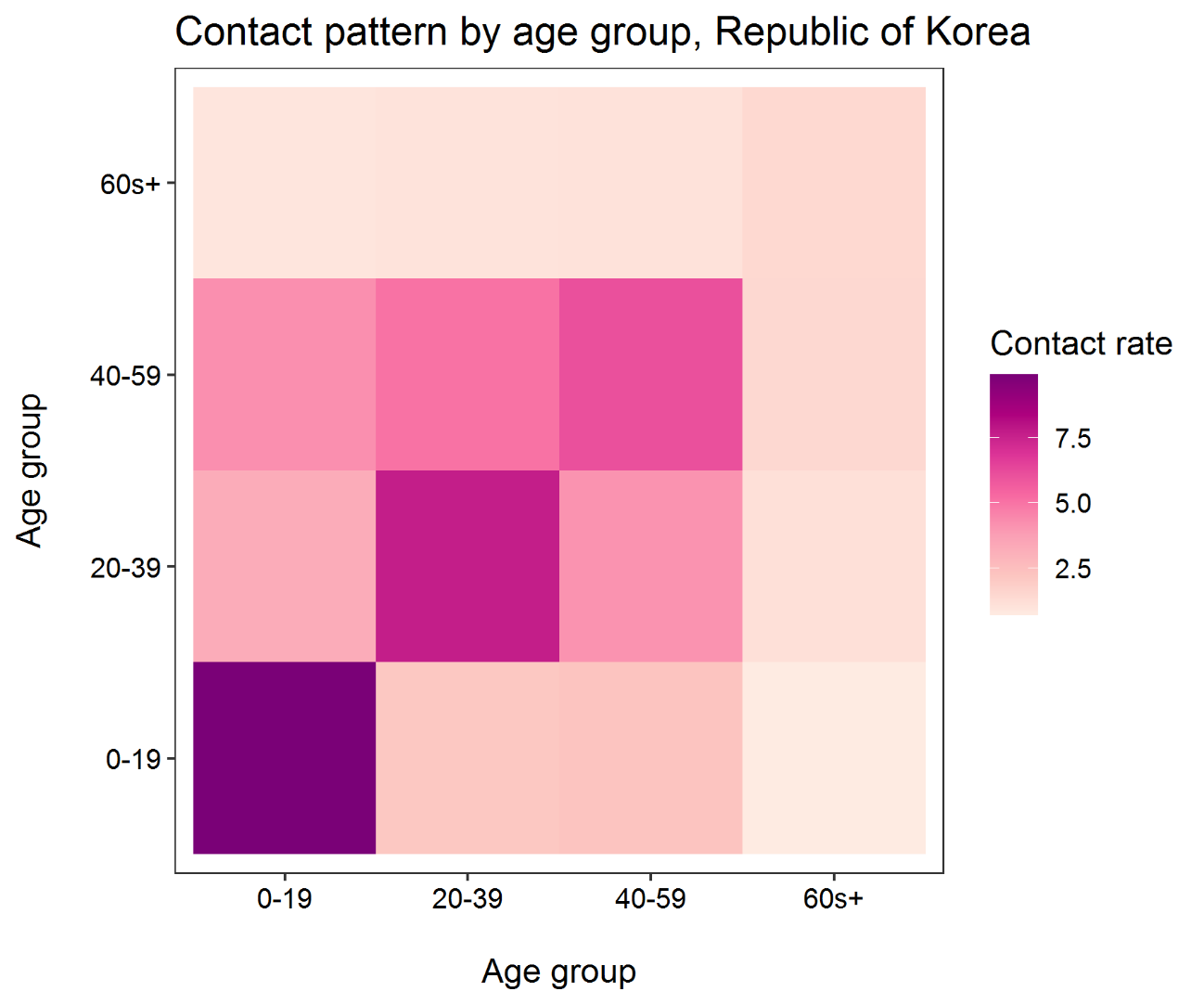
Figure S3. Rescaled contact pattern matrix of the Republic of Korea.**

Rescaled contact pattern matrix derived from the published contact matrix. The detailed method for the transformation is described in Section 2 (*Additional file*).

**
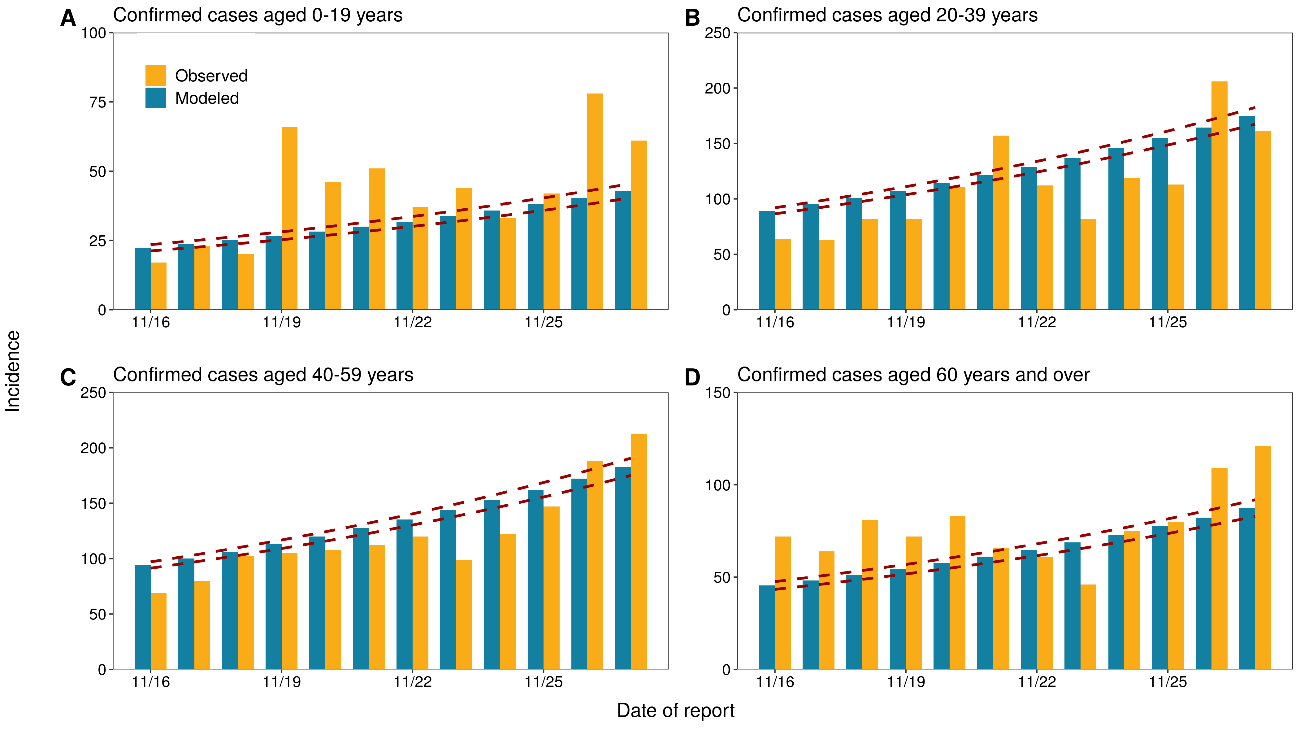
Figure S4. Comparison of observed and modelled incidence of COVID-19 by age group in the Republic of Korea from 16–27 November 2020**

Comparison between the observed and modelled incidence of COVID-19 in the Republic of Korea by four age groups: (**A**) 0–19, (**B**) 20–39, (**C**) 30–49, and (**D**) those aged 60 and over. Yellow bars represent the observed incidence in each age group, while blue bars are modelled incidence derived using the baseline model and empirically observed delay distribution from infection to report. Dashed red lines are 95% confidence intervals derived from the Laplace-approximate normal distribution.

**
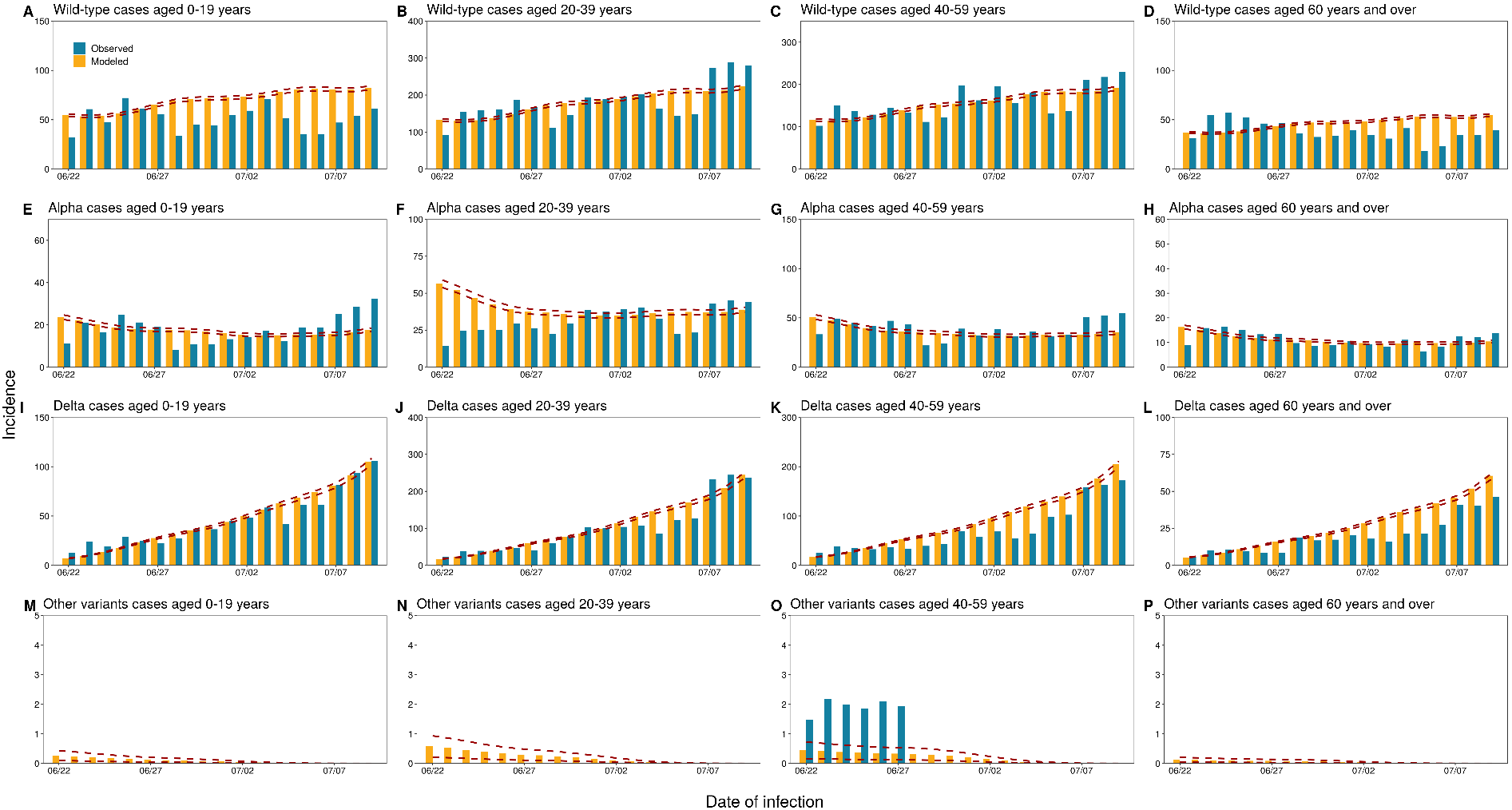
**

**Figure S5. Comparison of observed and modelled incidence of COVID-19 by age group and by SARS-CoV-2 variant in the Republic of Korea.**

Comparison between the observed and modelled incidence of COVID-19 in the Republic of Korea by four age group and by variant: (**A–D**) Wild-type variant, (**E–H**) Alpha variant, (**I–L**) Delta variant, and (**M–P**) the other SARS-CoV-2 variants. Yellow bars represent the observed reported case counts, whereas blue bars are modelled incidence derived using the baseline model and empirically observed delay distribution from infection to report. Dashed red lines are 95% confidence interval derived from the Laplace-approximate normal distribution.

**6. *Supplementary Information* Tables**

**Table S1. The details of the COVID-19 social distancing countermeasures by level in the Republic of Korea**

|  | Level 1 | Level 2 | Level 3 | Level 4 |
| --- | --- | --- | --- | --- |
| Transmission type | Not specified | Local transmission | Regional transmission | Nationwide transmission |
| Object | Containment | Limiting the size of gathering | Ban on gatherings | Ban on all outings |
| Implementing criteria | Less than 1 case per 100,000 population^*^ | More than 1 cases per 100,000 population^†^ | More than 2 cases per 100,000 population^†^ | More than 4 cases per 100,000 population^†^ |
| Personal gatherings | Not specified | Ban on gatherings of more than 9 persons | Ban on gatherings of more than 5 persons | Ban on gatherings of more than 5 persons |
| Events | Advance report is required when the event has more than 500 persons | Ban on evets of more than 100 persons | Ban on evets of more than 50 persons | Prohibiting all events |
| Assemblies | Ban on assemblies of more than 500 persons | Ban on assemblies of more than 100 persons | Ban on assemblies of more than 50 persons | Ban on assemblies of more than 2 persons |

* When the average of weekly incidence fulfills the criteria.

† When the average of weekly incidence fulfills the criteria more than 3 days a week.

**Table S2. Reconstructed next generation matrix of wild-type SARS-CoV-2 variant in the Republic of Korea**

|  | 0–19 | 20–39 | 40–59 | 60S+ |
| --- | --- | --- | --- | --- |
| 0–19 | 0.49 (0.48–0.51) | 0.16 (0.16–0.16) | 0.21 (0.21–0.22) | 0.05 (0.04–0.05) |
| 20–39 | 0.24 (0.23–0.24) | 0.86 (0.84–0.87) | 0.57 (0.55–0.57) | 0.11 (0.11–0.11) |
| 40–59 | 0.26 (0.26–0.27) | 0.48 (0.48–0.50) | 0.70 (0.70–0.73) | 0.12 (0.12–0.13) |
| 60S+ | 0.18 (0.16–0.18) | 0.29 (0.27–0.29) | 0.37 (0.35–0.37) | 0.36 (0.34–0.36) |
| $\mathcal{R}$ = 1.49 (1.47–1.50) | | | | |

**Table S3. Summary of parameters used in modeling and projection**

| Parameter | Description | Value |
| --- | --- | --- |
| Parameters for modelling and simulation | | |
| $\epsilon$ | Latent period | 3 days^14^ |
| $\sigma$ | Infectious period | 5 days^14^ |
| $\omega$ | Hospitalization period | 18 days (empirically observed) |
| $u_{i}$ | Relative susceptibility to infection for age group $i$ individuals | 0–19: 0.01 (95% CIs: 0.01–0.02)  20–39: 0.02 (95% CIs: 0.02–0.02)  40–59: 0.02 (95% CIs: 0.02–0.02)  60+: 0.05 (95% CIs: 0.04–0.05) |
| ${IFR}_{i}$ | Infection fatality risk for age group $i$ individuals | 0–19: 0.001  20–39: 0.02  40–59: 0.21  60+: 8.36^6^ |
| ${SR}_{i}$ | Risk of severity for age group $i$ individuals | 0–19: 0.001  20–39: 0.002  40–59: 0.01  60+: 0.09^5^ |
| $M_{ij}$ | Number of individuals in age group $j$ contacted by an individual in age group $i$ per day | Contact pattern matrix (Figure S2)^2^ |
| $k_{v}$ | Relative transmissibility of SARS-CoV-2 variants | Wild-type: reference (set as 1) ^*^  Alpha: 0.87 (95% CIs: 0.82–0.91)  Delta: 1.60 (95%CIs: 1.54–1.67)  The others: 0.29 (95% CIs: 0.11–0.47) ^†^ |
| $h$ | Reduction in the transmissibility of SARS-CoV-2 variants due to the enhanced social distancing countermeasures (compare to Level 1) | 0.89 (95% CIs: 0.87–0.91) |
| $l$ | Reduction in the transmissibility of SARS-CoV-2 variants due to Level 4 social distancing countermeasures implemented from 12 July 2021 | Varied (0.3, 0.35, and 0.4) |
| ${ve}_{v}$ | Vaccine effectiveness against infection | [1st dose]^§^  AZD1222: 50% / 30%^15^  JNJ-78436735^‡^: 50% / 30% (assumed)  BNT162b2 & mRNA-1273: 50% / 35%  [2nd dose] ^*^  AZD1222: 77% / 66%^15^  BNT162b2 & mRNA-1273: 95% / 88%^15^ |
| ${ve}_{H}$ | Vaccine effectiveness against hospitalization | 95%^13^ |

* A dominant circulating SARS-CoV-2 variant in November 2020 of Korea.

† All SARS-CoV-2 variants other than dominant variant, Alpha variant, and Delta variant.

^§^The former value reflects the vaccine effectiveness against the wild-type SARS-CoV-2 variant, whereas the latter refers to the Delta variant.

‡JNJ-78436735 vaccine requires only 1 shot. In our study, the effectiveness of JNJ-78436735 vaccine was assumed to be identical with that of AZD1222.

**7. References**

1. Ministry of Health and Welfare. Press release on COVID-19 in the Republic of Korea. http://ncov.mohw.go.kr/tcmBoardView.do?contSeq=360602 (2020).

2. Prem, K., Cook, A. R. & Jit, M. Projecting social contact matrices in 152 countries using contact surveys and demographic data. *PLoS Comput. Biol.* **13**, e1005697 (2017).

3. Röst, G. *et al.* Early Phase of the COVID-19 Outbreak in Hungary and Post-Lockdown Scenarios. *Viruses*  vol. 12 (2020).

4. Bubar, K. M. *et al.* Model-informed COVID-19 vaccine prioritization strategies by age and serostatus. *Science (80-. ).* **371**, 916 LP – 921 (2021).

5. National COVID-19 Vaccination Aadvisory Board in Koera. *Risk-benefit analysis of ChAdOx1 nCoV-19 vaccine about the issue of unusual blood clots with low blood platelets*. *Weekly Issue of Health and Disease* vol. 14 https://kdca.go.kr/board/board.es?mid=a20602010000&bid=0034&list_no=713111&act=view (2021).

6. Levin, A. T. *et al.* Assessing the age specificity of infection fatality rates for COVID-19: systematic review, meta-analysis, and public policy implications. *Eur. J. Epidemiol.* **35**, 1123–1138 (2020).

7. Ministry of Health and Welfare. Press release on COVID-19 in the Republic of Korea. https://www.korea.kr/news/policyBriefingView.do?newsId=156458445 (2021).

8. Linton, N. M. *et al.* Incubation Period and Other Epidemiological Characteristics of 2019 Novel Coronavirus Infections with Right Truncation: A Statistical Analysis of Publicly Available Case Data. *J. Clin. Med.* **9**, 538 (2020).

9. Davies, N. G. *et al.* Estimated transmissibility and impact of SARS-CoV-2 lineage B.1.1.7 in England. *Science (80-. ).* eabg3055 (2021) doi:10.1126/science.abg3055.

10. Nishiura, H., Linton, N. M. & Akhmetzhanov, A. R. Serial interval of novel coronavirus (COVID-19) infections. *Int. J. Infect. Dis.* (2020) doi:10.1016/j.ijid.2020.02.060.

11. Ministry of Health and Welfare. Press release on Level 4 social distancing in the Republic of Korea. https://www.mohw.go.kr/react/al/sal0301vw.jsp?PAR_MENU_ID=04&MENU_ID=0403&page=1&CONT_SEQ=366376 (2021).

12. Tregoning, J. S., Flight, K. E., Higham, S. L., Wang, Z. & Pierce, B. F. Progress of the COVID-19 vaccine effort: viruses, vaccines and variants versus efficacy, effectiveness and escape. *Nat. Rev. Immunol.* **21**, 626–636 (2021).

13. Chemaitelly, H. *et al.* mRNA-1273 COVID-19 vaccine effectiveness against the B.1.1.7 and B.1.351 variants and severe COVID-19 disease in Qatar. *Nat. Med.* **27**, 1614–1621 (2021).

14. Davies, N. G. *et al.* Age-dependent effects in the transmission and control of COVID-19 epidemics. *Nat. Med.* **26**, 1205–1211 (2020).

15. Baden, L. R. *et al.* Efficacy and Safety of the mRNA-1273 SARS-CoV-2 Vaccine. *N. Engl. J. Med.* **384**, 403–416 (2020).
